# A Novel Energy-Efficient MAC Aware Data Aggregation Routing in Wireless Sensor Networks^#^

**DOI:** 10.3390/s90301518

**Published:** 2009-03-04

**Authors:** Frank Yeong-Sung Lin, Hong-Hsu Yen, Shu-Ping Lin

**Affiliations:** 1 Dept. of Information Management, National Taiwan University / No.1, Sec. 4, Roosevelt Rd., Taipei City 106, Taiwan (R.O.C.); E-Mails: yslin@im.ntu.edu.tw; r92725018@ntu.edu.tw; 2 Dept. of Information Management, Shih Hsin University / No. 1, Lane17, Sec.1, Mu-Cha Rd., Taipei City 116, Taiwan (R.O.C.)

**Keywords:** Data aggregation, MAC aware data aggregation routing, CSMA/CA, Lagrangean relaxation, wireless sensor networks

## Abstract

Embedding data-aggregation capabilities into sensor nodes of wireless networks could save energy by reducing redundant data flow transmissions. Existing research describes the construction of data aggregation trees to maximize data aggregation times in order to reduce data transmission of redundant data. However, aggregation of more nodes on the same node will incur significant collisions. These MAC (Media Access Control) layer collisions introduce additional data retransmissions that could jeopardize the advantages of data aggregation. This paper is the first to consider the energy consumption tradeoffs between data aggregation and retransmissions in a wireless sensor network. By using the existing CSMA/CA (Carrier Sense Multiple Access with Collision Avoidance) MAC protocol, the retransmission energy consumption function is well formulated. This paper proposes a novel non-linear mathematical formulation, whose function is to minimize the total energy consumption of data transmission subject to data aggregation trees and data retransmissions. This solution approach is based on Lagrangean relaxation, in conjunction with optimization-based heuristics. From the computational experiments, it is shown that the proposed algorithms could construct MAC aware data aggregation trees that are up to 59% more energy efficient than existing data aggregation algorithms.

## 1. Introduction

**Figure 1.**
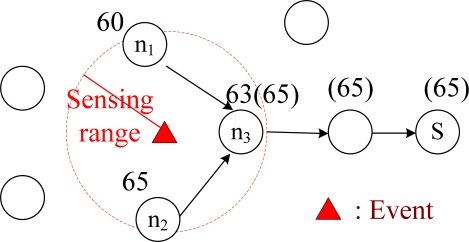
Data aggregation in MAX.

Wireless sensor networks (WSNs) have been blooming recently, which can probe and collect environmental information, such as temperature, atmospheric pressure, and irradiation to provide ubiquitous sensing, computing, and communication capabilities. A WSN has two important and interesting characteristics that are different from traditional wireless networks. First, after the event occurs, multiple sensors nodes (denoted as *data source* nodes) around this event will sense the event, and then send the data back to one sensor node (denoted as *sink* node). Hence, communication mode in WSN occurs from multiple data source nodes to one data sink node. This is a type of *multipoint-to-point*, rather than the traditional point-to-multipoint (i.e. multicast) communication in wireless networks. For example, Figure 1 shows a data aggregation tree from three data source nodes to one sink. This data aggregation tree is a type of reverse-multicast tree. Second, energy saving is possible at the nodes on the data aggregation tree because intermediate nodes on the data aggregation tree could receive redundant data from the data source nodes. In order to avoid transmitting useless, redundant data back to the sink, the intermediate nodes could save energy by collecting and processing data before transmission and prevent disconnected networks due to rapid energy depletion of sensors. This type of *data aggregation* capability has been put forward as particularly useful for routing, in terms of energy consumption in WSN [2].

There are several data aggregation schemes, and in addition to reducing redundant transmissions, other aggregation schemes could compute maximum values (MAX), minimum values (MIN), or summations (SUM) of the collected data. For example, in Figure 1, an event in sensing range of data source nodes *n_1_*, *n_2_*, and *n_3_* is probed for temperature (60, 65, and 63°F, respectively), and the MAX temperature is then sent back to the sink node *S*. If node *n_3_* could aggregate (i.e. MAX = 65°F) these data before returning it to the sink, the total number of transmission times for node *n_3_* could be reduced from three to one.

Since it is almost impossible to replace the battery in a sensor node, power efficient communication in WSN plays a crucial role. In data aggregation routing, the key issue is how to construct the reverse multicast tree in such a way to save the total energy consumption. Most existing research literatures construct the tree by only considering the data aggregation aspect [2,6]. The basic idea of these data aggregation aspect algorithms is trying to maximize the times of aggregation to reduce the number of transmissions. However, there remains one issue important to the construction of a data aggregation tree, the *MAC layer retransmission* issue.

**Figure 2.**
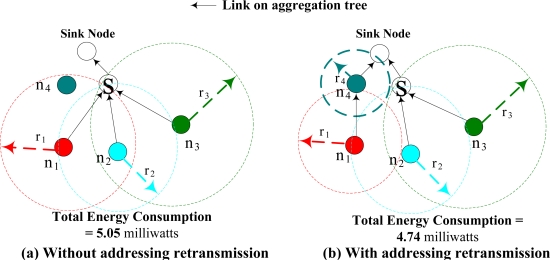
Tradeoff between retransmission and data aggregation.

In WSNs, any sensor nodes within another’s transmission range trying to transmit simultaneously would result in collision. In addition, two nodes that are not within each other’s transmission range trying to simultaneously transmit to the same node might also incur collision. This is well known as the hidden-node problem. Because of hidden-node problem, the interference range is larger than the transmission range in wireless communications. In Figure 2(a), shows that even though the transmission radius of nodes *n_1_* and *n_3_* do not overlap, collision still occurs at the receiver (node *S*) when they transmit at the same time. When collision occurs, *retransmission* is required to ensure the data is successfully received. These retransmissions incur additional energy consumption, which will jeopardize the advantages of data aggregation. Data retransmission times are determined by the total number of sensor nodes whose transmission radius covers the receiver (or equivalent to the total number of sensor nodes within each other’s interference range). In other words, the more flows are aggregated, the higher the probability that the senders will incur data retransmission. Hence, there is a tradeoff between data aggregation and retransmission. Good data aggregation tree should address data aggregation and MAC layer retransmission at the same time.

Figure 2 gives an illustrative example to show the tradeoff between the data aggregation and retransmission, where nodes *n_1_*, *n_2_*, and *n_3_* are the data source nodes. Without considering data collision, the optimal aggregation tree is as shown in Figure 2(a). Note that when an intermediate node aggregates more data, a greater number of collisions would occur at the intermediate nodes, which results in additional energy consumption. Node *S*, the receiver of the three children nodes, will suffer significant collisions that results in more retransmission times. With considered collision effects, a more energy efficient data aggregation tree is as shown in Figure 2(b). In this figure, by reducing the transmission radius of node *n_1_*, and change its routing assignment to node *n_4_*, the total energy consumption could be reduced. Even though there is extra energy consumption at node *n_4_*, there are only two children nodes at node *S,* and thus, the retransmission times caused by collision could be significantly reduced. Hence, the energy consumption associated with retransmission from collisions should be carefully addressed in WSN. This example also shows that a good tradeoff between data aggregation and retransmission is facilitated by intelligent transmission radius and routing assignments. The energy consumption function (including transmission power and retransmission power), as shown in Figure 2, is calculated by its objective function (IP), as described in Section 3.

This paper discusses the impacts of retransmission on data aggregation, and proposes a MAC aware energy efficient data aggregation algorithm to consider a tradeoff between the benefits of data aggregating and data retransmission costs in WSN. To the best of our knowledge, there is no literature that addresses the cross-layer (layer 2 and layer 3) MAC aware data aggregation routing algorithm in WSNs. This paper proposes an optimization-based heuristic algorithm to solve the MAC aware energy-efficient data aggregation routing problems (MAC-DAR) based on the CSMA/CA protocol in WSNs. The problem is first formulated as a nonlinear programming problem, where the objective function is to minimize total energy consumption from data transmissions and retransmissions. The Lagrangean relaxation scheme in conjunction with the optimization-based heuristic algorithm is proposed to solve this problem. From the computational experiments, the proposed solution approach outperforms the conventional non-MAC aware data aggregation heuristics. In addition, the proposed nonlinear programming formulation for the MAC-DAR problem is based on the existing CSMA/CA protocol, and thus, our algorithm could be deployed in the wireless sensor network, without the necessity of modifying the MAC protocol in WSNs. In summary, besides better solution quality, our proposed approach could be easily deployed in WSNs without changing the existing CSMA/CA protocol.

The remainder of this paper is organized as follows. Section 2, surveys existing related works on data aggregation routing and MAC layer protocols in WSNs. In Section 3, mathematical formulation of the MAC-DAR in WSNs is proposed. In Section 4, solution approaches, as based on Lagrangean relaxation are presented. In Section 5, heuristics are developed for calculating a good primal feasible solution. In Section 6, computational results are reported. Finally, Section 7 concludes this paper.

## 2. Related Works

Existing researches have been conducted to address pure data aggregation routing problem in WSN. In [2], they devise three interesting suboptimal aggregation heuristics, Shortest Paths Tree (SPT), Center at Nearest Source (CNS), and Greedy Incremental Tree (GIT) for data centric routing problems. In [6], mathematical formulations for data aggregation problem in WSN are well formulated, and an optimization-based heuristic algorithm is then proposed to tackle the problem. In [5], they address latency issues in constructing a minimum energy aggregation tree, and propose the CCA algorithm, which includes the basic idea of a balanced tree to simultaneously minimize energy and latency issues.

Several papers have discussed MAC layer protocol in ad-hoc and sensor networks [7–9]. X. H. Lin [9] enhanced the standard IEEE 802.11 MAC protocol by improving the handshake and power control mechanisms. W. Ye [7,8] reviewed several MAC protocols, and discussed design tradeoffs on energy efficiency and data transmissions. W. Ye proposed S-MAC protocol to fit the energy-efficient requirements for sensor networks, which is also a variation of a CSMA-like protocol that needs extra messages for transmitting data.

Several works have proposed cross-layer algorithms to deal with retransmission issues caused by collisions in wireless sensor networks. In [10], instead of dealing with the retransmission issue directly, they assign sensor nodes within each other’s interference range, which have different channels to circumvent collision problems. They proposed integrated channel assignments and data aggregation routing algorithms in WSNs. In [11,12], the authors proposed an interesting MAC layer anycasting mechanism and randomized waiting at the application layer, to facilitate data aggregation spatially and temporally in structure-free sensor networks. They address the collision problem by proposing a modified CSMA/CA protocol and randomized waiting scheme to reduce the number of retransmissions.

## 3. Problem Formulation

A MAC-DAR in WSNs is modeled as a graph, in which sensors are represented as nodes, and the arc connecting the two nodes indicates that one node is within the other’s transmission radius. The definitions of notations adopted in the formulation are listed below.

First, the given parameters are shown as follows:

**Table:** 

*N*	The set of all sensor nodes
*P_sq_*	The set of all candidate paths that connect data source node *s* to sink node *q*
*S*	The set of all data source nodes
*h*	Longest distance of shortest path to reach the farthest data source node
*M*	An arbitrary large number
*δ_p(n,k)_*	The indicator function, which is 1 if the link from node *n* to node *k* is on path *p*, and 0 otherwise
*d_nk_*	Euclidean distance between node *n* and node *k*
*t_data_*	Transmission time for transmitting a data packet
*RTS*	Transmission time for RTS frame
*SIFS*	Short inter-frame space time
*θ*	Maximum propagation delay for transmitting data packet
*Q*	The sink node
*R_n_*	The set of all possible transmission radii that node *n* can adopt, which is a discrete set
*e_n_* (*r_n_*)	Energy consumption function of node *n* per unit time, which is a function of the sensor’s transmission radius
*T*	The largest number of retransmission times

Then, the decision variables are shown below.

**Table:** 

*x_sp_*	1 if data source node *s* uses path *p* to reach sink node *q*, and 0 otherwise
*y_(n,k)_*	1 if the link from node *n* to node *k* is on the tree, and 0 otherwise
*r_n_*	Transmission radius of the node *n*
*z_nk_*	1 if node *k* is covered within transmission radius of node *n*, and 0 otherwise
*c_nk_*	Retransmission times of node *n* to transmit data to node *k*

Please note that we do not have to generate all candidate paths that connect data source node *s* to sink node *q* (i.e., *P_sq_*). Section 4 will explain by using Lagrangean multipliers as the link arc weight (in Subproblem 2), *x_sp_* will be associated with the minimum-weight path by using the shortest path algorithm for each data source node *s*.

An analysis of retransmission times is conducted as follows. First, it assumes that each sensor node is equipped with a CSMA/CA compatible transceiver, each transmission conforms to a Geometric distribution, and each sensor node generates data packets that follow a Poisson distribution at a certain rate of λ. Successful transmission of data from sender to receiver depends on the number of senders whose transmission radius covers the receiver. By considering receiver side collisions, in terms of communication radii of sensor nodes, the hidden-node problem is implicitly contemplated. In the CSMA/CA protocol, when a sender wants to transmit a packet to a receiver, it will first issue an RTS control frame and wait for a CTS frame from the receiver to ensure that the channel be free [4]. According to the CSMA/CA protocol, the time interval between RTS and CTS is no larger than a short inter-frame spacing (*SIFS*) time. Let the propagation delay from sender to receiver be *θ,* and turnaround time be *2θ*. The overall contention period is (*RTS* + *SIFS + 2θ*). Then the average retransmission time from node *n* to node *k* (i.e., *c_nk_*) is as follows:

(0) $$\mathrm{Average}\;\mathrm{Retransmission}\;\mathrm{Time}s_{\left(n,k\right)}=\frac{1}{p_{\mathrm{success}(n,k)}}=\frac{1}{e^{-\lambda (\underline{\mathrm{RTS}}+\underline{\mathrm{SIFS}}+2\theta )\sum_{j\in N}z_{\mathrm{jk}}}}.$$

∑_*j*∈*N*_ *z_jk_* calculates the total number of senders whose transmission radius covers node *k*. The meaning of (0) is the mean value of the Geometric distribution, where the successful transmission probability, say *p_success_*, is that no data transmissions are occurring at any node whose transmission radius covers receiver node *k* within the contention period (*RTS+SIFS+2θ*).

The MAC-DAR problem in WSNs is then formulated as the following nonlinear optimization problem (IP).

(IP) $$Z_{\mathrm{IP}}=\text{min}\sum_{n\in N}\left(t_{\mathrm{data}}+(\underline{\mathrm{RTS}}\cdot \sum_{k\in N}c_{\mathrm{nk}})\right)\cdot e_{n}(r_{n})$$

subject to:

(1) $$\begin{array}{ll} \sum_{p\in P_{\mathrm{sq}}}x_{\mathrm{sp}}\;\delta_{p(n,k)}\leq y_{\left(n,k\right)} & \forall n,\;k\in N,\;s\in S \end{array}$$

(2) $$\sum_{n\in N}\sum_{k\in N}y_{\left(n,k\right)}\geq \text{max}\{h,|S|\}$$

(3) $$\begin{array}{ll} \sum_{s\in S}\sum_{p\in P_{sq}}x_{\mathrm{sp}}\;\delta_{p(n,k)}\leq \left|S\right|\cdot y_{\left(n,k\right)} & \forall n,k\in N \end{array}$$

(4) $$\begin{array}{ll} \sum_{p\in P_{\mathrm{sq}}}x_{\mathrm{sp}}=1 & \forall s\in S \end{array}$$

(5) $$\begin{array}{ll} \sum_{k\in N}y_{\left(n,k\right)}\leq 1 & \forall n\in N \end{array}$$

(6) $$\begin{array}{ll} \frac{r_{n}-d_{\mathrm{nk}}}{M}\leq z_{\mathrm{nk}} & \forall n,k\in N \end{array}$$

(7) $$\begin{array}{ll} z_{\mathrm{nk}}d_{\mathrm{nk}}\leq r_{n} & \forall n,k\in N \end{array}$$

(8) $$\begin{array}{ll} y_{\left(n,k\right)}\leq z_{\mathrm{nk}} & \forall n,k\in N \end{array}$$

(9) $$\begin{array}{ll} c_{\mathrm{nk}}\geq \frac{e^{-(1-y_{\left(n,k\right)})M}}{e^{-\lambda (\underline{\mathrm{RTS}}+\underline{\mathrm{SIFS}}+2\theta )\sum_{j\in N}z_{\mathrm{jk}}}} & \forall n,k\in N \end{array}$$

(10) $$\begin{array}{ll} x_{\mathrm{sp}}=0\;\;\text{or}\;\;1 & \forall s\in S,p\in P_{\mathrm{sq}} \end{array}$$

(11) $$\begin{array}{ll} y_{\left(n,k\right)}=\;0\;\text{or}\;1 & \forall n,k\in N \end{array}$$

(12) $$\begin{array}{ll} z_{\mathrm{nk}}=\;0\;\text{or}\;1 & \forall n,k\in N \end{array}$$

(13) $$\begin{array}{ll} r_{n}\in R_{n} & \forall n\in N \end{array}$$

(14) $$\begin{array}{ll} r_{n}\neq 0 & \forall n\in S \end{array}$$

(15) $$\begin{array}{ll} c_{\mathrm{nk}}\in \{0,\;1,\;2,....,T\} & \forall n,k\in N\; \end{array}$$

The objective function of (IP) is to minimize total energy consumption, where $\sum_{n\in N}t_{\mathrm{data}}\cdot e_{n}(r_{n})$ captures the energy consumption from data transmission; and $\sum_{n\in N}\left(\underline{\mathrm{RTS}}\cdot \sum_{k\in N}c_{\mathrm{nk}}\right)\cdot e_{n}(r_{n})$ captures the energy consumption from retransmission. In conjunction with the objective function, three sets of constraints (*data aggregation tree, transmission coverage,* and *retransmission times*) are enforced in Problem (IP) to give the MAC-DAR problem.

### A. Data aggregation tree constraint

The basic idea of this set of Constraints, are to ensure that the union of all routing paths, from data source nodes to sink shall be a data aggregation tree. Recall that a data aggregation tree is a reverse-multicast tree, which is a multicast tree rooted at the sink node, but with opposite transmission directions. The data aggregation tree properties are enforced by Constraints (1) to (5). Constraint (1) requires that if path *p* is selected for source node *s* to reach sink node *q*, the path must be on the tree. This constraint also enforces that if links (*n*, *k*) on path *p* are adopted by source node *s* to reach the sink node, then *y_(n,k)_* must be 1. Constraints (2) and (11) require that the total number of links on an aggregation tree is at least the maximum of *h* and the cardinality of *S*. Both *h* and |*S*| are legitimate lower bounds of the total number of links on an aggregation tree, and they could be calculated in advance [3]. According to [3], introducing Constraint (2) will significantly improve solution quality. The left-hand term of Constraint (3) calculates the number of paths that are destined for the sink node, and pass through link (*n, k*) on the aggregation tree. The right-hand term of Constraint (3) is at most |*S*|. When the union of paths destined for a sink node contains a cycle, and this cycle contains link (*n, k*), then Constraint (3) would not be satisfied because there would be too many paths passing through this link. In other words, Constraint (3) enforces the union of paths that do not contain a cycle [6]. Constraints (4) and (10) require that any data source adopts only one routing path destined for the sink node. Constraint (5) is the outgoing link constraint. All intermediate nodes on the aggregation tree should have only one outgoing link. For example, in Figure 1, each node on the data aggregation tree has only one outgoing link to the sink node. In summary, Constraints (1)–(5), (10), and (12) enforce that the union of all routing paths shall be a data aggregation tree.

### B. Transmission coverage constraint

The basic idea of this set of constraints are to ensure that if a node *k* is covered within the transmission radius of node *n*, then the distance between node *n* and node *k* must be smaller than the transmission radius of node *n*. Because *M* is a very large number, on the left hand side of Constraint (6), if *r_n_* > *d_nk_* (i.e., node *k* is within the transmission radius of node *n*), then $0<\frac{r_{n}-d_{\mathrm{nk}}}{M}<1$. This will force *z_nk_* to be equal to 1. On the other hand, if *r_n_* > *d_nk_* then $\frac{r_{n}-d_{\mathrm{nk}}}{M}\leq 0$, then, *z_nk_* could be equal to 0. Constraint (7) enforces that if node *k* is covered within the transmission radius of the node *n*, then the transmission radius of node *n* must be larger than the distance between nodes *n* and *k*. Hence, Constraints (6) and (7) specify the transmission coverage constraints for decision variables *r_n_* and *z_jk_*. Then ∑_*j*∈*N*_ *z_jk_*, which is used in Equation (0), calculates the total number of senders whose transmission radius covers the node *k*. Constraint (8) relates decision variable *y_(n,k)_* to *z_nk_*. When *y_(n,k)_* equals to 1, it will force *z_nk_* to be 1.

Constraint (13) restricts that the set of possible transmission radii that node *n* can adopt is a discrete and finite set. Constraint (14) ensures that each data source node turns on its transmission radius. The transmission radius of each source node cannot be 0.

### C. Retransmission time constraints

The basic idea of this set of constraints are to calculate the retransmission times of node *n* to transmit data to node *k*, where the retransmission times are determined by the total number of nodes on the data aggregation tree, whose transmission radius covers node *k*. Constraint (9) calculates the retransmission times of node *n* to transmit data to node *k*. Since only the sensor nodes on the aggregation tree need to calculate retransmission times, when *y_(n,k)_* = 1, the right side of Constraint (9) is the same as Equation (0), i.e., to enforce the retransmission times (i.e., *c_nk_*) and should be at least the average retransmission times. When *y_(n,k)_* = 0, the right side of Constraint (9) is zero, it implies that there is no retransmission time constraint. Constraint (15) is an integer constraint of retransmission times.

In order to make the problem (IP) tractable, a natural logarithm is used on both sides of Constraint (9) for applying the Lagrangean relaxation schemes,

$$\text{ln}(c_{\mathrm{nk}})\geq \text{ln}\left(\frac{e^{-(1-y_{\left(n,k\right)})M}}{e^{-\lambda (\mathrm{RTS}+\mathrm{SIFS}+2\theta )\sum_{j\in N}z_{\mathrm{jk}}}}\right)\Rightarrow \text{ln}(c_{\mathrm{nk}})\geq \lambda \;(\mathrm{RTS}+\mathrm{SIFS}+2\theta )\sum_{j\in N}z_{\mathrm{jk}}-M+{\mathrm{My}}_{\left(n,k\right)}$$

Hence, Constraint (9) becomes

(9) $$\begin{array}{ll} \text{ln}(c_{\mathrm{nk}})\geq \;\lambda \;(\mathrm{RTS}+\mathrm{SIFS}+2\theta )\sum_{j\in N}z_{\mathrm{jk}}-M+{\mathrm{My}}_{\left(n,k\right)} & \forall n,k\in N \end{array}$$

## 4. Lagrangean Relaxation

The algorithm structure is based upon Lagrangean relaxation. In (IP), by introducing Lagrangean multiplier vectors *u^1^*, *u^2^*, *u^3^*, *u^4^*, *u^5^*, and *u^6^*, Constraints (1), (3), (6), (7), (8), and (9) are dualized to obtain the following Lagrangean relaxation problem (LR).

(LR) $$\begin{array}{l} Z_{D}=\text{min}\;\sum_{n\in N}\left(t_{\text{data}}+(\underline{\mathrm{RTS}}\cdot \sum_{k\in N}c_{\mathrm{nk}})\right)\cdot e_{n}(r_{n})+\sum_{n\in N}\sum_{k\in N}\sum_{s\in S}u_{\mathrm{nks}}^{1}\left(\sum_{p\in P_{\mathrm{sq}}}x_{\mathrm{sp}}\delta_{p(n,k)}-y_{\left(n,k\right)}\right)+\sum_{n\in N}\sum_{k\in N}u_{\mathrm{nk}}^{2}\left(\sum_{s\in S}\sum_{p\in P_{\mathrm{sq}}}x_{\mathrm{sp}}\delta_{p(n,k)}-|S|y_{\left(n,k\right)}\right)+\\ \sum_{n\in N}\sum_{k\in N}u_{\mathrm{nk}}^{3}(r_{n}-d_{\mathrm{nk}}-{\mathrm{Mz}}_{\mathrm{nk}})+\sum_{n\in N}\sum_{k\in N}u_{\mathrm{nk}}^{4}(z_{\mathrm{nk}}d_{\mathrm{nk}}-r_{n})+\sum_{n\in N}\sum_{k\in N}u_{\mathrm{nk}}^{5}\left(y_{\left(n,k\right)}-z_{\mathrm{nk}}\right)+\sum_{n\in N}\sum_{k\in N}u_{\mathrm{nk}}^{6}(\lambda (\underline{\mathrm{RTS}}+\underline{\mathrm{SIFS}}+2\theta )\sum_{j\in N}z_{\mathrm{jk}}-\\ M(1-y_{\left(n,k\right)})-\text{ln}(c_{\mathrm{nk}})) \end{array}$$

subject to:

(16) $$\sum_{n\in N}\sum_{k\in N}y_{\left(n,k\right)}\geq \text{max}\{h,|S|\}$$

(17) $$\begin{array}{ll} \sum_{p\in P_{\mathrm{sq}}}x_{\mathrm{sp}}=1 & \forall s\in S \end{array}$$

(18) $$\begin{array}{ll} \sum_{k\in N}y_{\left(n,k\right)}\leq 1 & \forall n\in N \end{array}$$

(19) $$\begin{array}{ll} x_{\mathrm{sp}}=\;0\;\text{or}\;1 & \forall s\in S,p\in P_{\mathrm{sq}} \end{array}$$

(20) $$\begin{array}{ll} y_{\left(n,k\right)}=\;0\;\text{or}\;1 & \forall n,k\in N \end{array}$$

(21) $$\begin{array}{ll} z_{\mathrm{nk}}=\;0\;\text{or}\;1 & \forall n,k\in N \end{array}$$

(22) $$\begin{array}{ll} r_{n}\in R_{n} & \forall n\in N \end{array}$$

(23) $$\begin{array}{ll} r_{n}\neq 0 & \forall n\in S \end{array}$$

(24) $$\begin{array}{ll} c_{\mathrm{nk}}\in \{0,\;1,\;2,....,T\} & \forall n,k\in N \end{array}.$$

(LR) can be decomposed into four independent subproblems.

Subproblem 1: for *y*_(*n,k*)_
  (SUB1) $$\text{min}\;\sum_{n\in N}\sum_{k\in N}\left(u_{\mathrm{nk}}^{5}+u_{\mathrm{nk}}^{6}M-u_{\mathrm{nk}}^{2}|S\right|\;-\sum_{s\in S}u_{\mathrm{nks}}^{1})y_{\left(n,k\right)}$$
  subject to (16), (18) and (20).
Subproblem 2: for *x_sp_*
  (SUB2) $$\text{min}\;\sum_{n\in N}\sum_{k\in N}\sum_{s\in S}\sum_{p\in P_{\mathrm{sq}}}(u_{\mathrm{nks}}^{1}+u_{\mathrm{nk}}^{2})\;x_{\mathrm{sp}}\;\delta_{p(n,k)}$$
  subject to (17) and (19).
Subproblem 3: for *r_n_* and *c_nk_*
  (SUB3) $$\text{min}\;\sum_{n\in N}e_{n}(r_{n})\cdot t_{\mathrm{data}}+\underline{\mathrm{RTS}}\sum_{n\in N}\sum_{k\in N}e_{n}(r_{n})\cdot c_{\mathrm{nk}}+\sum_{n\in N}\sum_{k\in N}(u_{\mathrm{nk}}^{3}-u_{\mathrm{nk}}^{4})r_{n}-\sum_{n\in N}\sum_{k\in N}u_{\mathrm{nk}}^{6}\;\text{ln}(c_{\mathrm{nk}})$$
  subject to (22), (23) and (24).
Subproblem 4: for *z_nk_*
  (SUB4) $$\text{min}\;\sum_{n\in N}\sum_{k\in N}\left(u_{\mathrm{nk}}^{4}d_{\mathrm{nk}}-u_{\mathrm{nk}}^{3}M-u_{\mathrm{nk}}^{5}+\lambda (\underline{\mathrm{RTS}}+\underline{\mathrm{CTS}}+2\theta )\sum_{j\in N}u_{\mathrm{jk}}^{6}\right)\;z_{\mathrm{nk}}$$
  subject to (21).

The proposed algorithm for solving (SUB1) is described as follows.

**Step 1.** For each link (*n*,*k*) compute the coefficient $u_{\mathrm{nk}}^{5}+u_{\mathrm{nk}}^{6}M-u_{\mathrm{nk}}^{2}|S|-\sum_{s\in S}u_{\mathrm{nk}}^{1}$ for each *y_(n,k)_*.
**Step 2.** For all outgoing links of node *n*, find the smallest coefficient. If the smallest coefficient is negative, then set the corresponding *y_(n,k)_* as 1, and the other outgoing links *y_(n,k)_* as 0, otherwise set all outgoing links *y_(n,k)_* as 0. Repeat step 2 for all nodes.
**Step 3.** If the total number of *y_(n,k)_*, whose value is 1 (denoted as *τ*) are smaller than *max*{*h*, |S|}*,* then first let each *y_(n,k)_* whose corresponding coefficient is negative be 1. Second, assign the (*max*{*h*, |S|} −*τ*) number of *y_(n,k)_* to be 1 whose corresponding coefficients are the smallest positive values. Third, let the remaining *y_(n,k)_* be 0.

The computational complexity of above algorithm is *O*(*|N|^2^*).

(SUB2) can be further decomposed into |*S*| independent shortest path problems with nonnegative arc weight whose value is $u_{\mathrm{nks}}^{1}+u_{\mathrm{nk}}^{2}$. For each shortest path problem, it can be effectively solved by Dijkstra’s algorithm. The computational complexity of Dijkstra’s algorithm is *O*(*|N|^2^*) for each data source node.

(SUB3) can be optimally solved by exhaustively searching the combination of radius *r_n_* and *c_nk_*. The computational complexity of (SUB3) is *O*(|*R_n_*|*T*) for each node *n*.

In (SUB4), if the corresponding coefficient $u_{\mathrm{nk}}^{4}d_{\mathrm{nk}}-u_{\mathrm{nk}}^{3}M-u_{\mathrm{nk}}^{5}+\lambda (\mathrm{RTS}+\mathrm{CTS}+2\theta )\sum_{j\in N}u_{\mathrm{jk}}^{6}$ of link (*n*, *k*) is negative then set *z_nk_* to be 1, otherwise 0. The computational complexity of (SUB4) is *O*(1) for each link (*n*, *k*).

According to the algorithms proposed above, the Lagrangean relaxation problem can be effectively and optimally solved. Based on the weak Lagrangean duality theorem, *Z_D_(u^1^,u^2^,u^3^,u^4^,u^5^,u^6^)* is a lower bound of *Z_IP_*. The tightest lower bound is calculated by using the subgradient method [1].

## 5. Obtaining Primal Feasible Solutions

It is noted that solutions to the problem (LR) may not be feasible for the primal problem (IP), because six constraints are relaxed to the objective function. This paper proposes an optimization-based integrated primal feasible algorithm, called *LGR-Primal*, which jointly address data aggregation and retransmission to obtain primal feasible solutions to the problem (IP). The information in problem (LR) provides useful information to obtain good primal feasible solutions. In LGR-Primal, the information from the Lagrangean relaxation (the solutions to the dual problem and the Lagrangean multipliers) is used to optimize the tradeoff between data aggregation and retransmission.

LGR-Primal is presented in Algorithm 1; it identifies the routing path (i.e., *x_sp_*) for each data source node, and then the data aggregation tree is obtained by unifying all the routing paths from each data source node to the sink. In order to obtain an energy efficient data aggregation tree, the link arc weight assignment optimizes the tradeoff between data aggregation and retransmission.

When the routing path *x_sp_* of each data source node *s*, which is returning to the sink node, is determined, then the selected links (i.e., *y_(n,k)_*) on the data aggregation tree could also be determined. In addition, the transmission radius (i.e., *r_n_*) of each node could also be determined to cover the termination node for all selected links on the data aggregation tree. After the transmission radius of each node is determined, then the coverage decision variable *z_nk_* could also be determined. Finally, the value of retransmission time *c_nk_* could also be determined to satisfy Constraint (9).

In Step 1 of Algorithm 1, the first term of the arc weight assignment is energy consumption for link (*n*, *k*), *e_nk_* (*d_nk_*), which captures the minimum transmission power to select link (*n*, *k*) on the data aggregation tree. It is worth noting that, the physical meaning of the Lagrangean multiplier is the violating cost of the associated constraint. The second term, $u_{\mathrm{nk}}^{2}$, captures the penalty cost for violating the tree constraint. The third and forth terms, $u_{\mathrm{nk}}^{3}$ and $u_{\mathrm{nk}}^{4}$, capture penalty costs for violating transmission coverage constraints. The fifth and sixth terms, $u_{\mathrm{nk}}^{5}$ and $u_{\mathrm{nk}}^{6}$, capture the penalty costs for violating retransmission time constraints. Hence, based on this link arc weight assignment, this paper attempts to identify the minimum transmission power data aggregation tree by considering the extra costs from retransmissions.

The computational complexity of Algorithm 1 at Step 2 is *O*(|*S*||*N*|^2^). At Steps 3 and 4, when computing the retransmission power of the objective function, it requires calculation of the total number of other nodes, whose transmission radius covers node *k* (Equation (0)), in order to determine the retransmission times *c_nk_* from node *n* to node *k*. Therefore, the computational complexity is *O*(|*N*|^3^). Hence, the computational complexity of the LGR-Primal algorithm should be *O*(|*N*|^3^). However, if each sensor node *k* is equipped with GPS, which enables it to know their neighboring sensors nodes, then to determine the retransmission times of *c_nk_* from node *n* to node *k* would only need to calculate the *k*’s neighboring nodes (instead of all the other sensor nodes in the WSN) whose transmission radius covers node *k*. In this case, the computational complexity for Steps 3 and 4 would only be *O*(|*N*|^2^). Then the computational complexity of the LGR-Primal algorithm should be *O*(|*S*||*N*|^2^). This makes this algorithm scalable to a large scale WSN.

**Algorithm 1.** LGR-Primal Algorithm.

**Step 1) Assign** the arc weight of the each link (*n*, *k*) as $e_{\mathrm{nk}}\left(d_{\mathrm{nk}}\right)+u_{\mathrm{nk}}^{2}+u_{\mathrm{nk}}^{3}+u_{\mathrm{nk}}^{4}+u_{\mathrm{nk}}^{5}+u_{\mathrm{nk}}^{6}$. **Step 2) Perform** a Dijkstra’s shortest path algorithm to identify the routing path (i.e., *x_sp_*) from each data source node *s* to the sink node. **Step 3) Determine** the other decision variables (*y_(n,k)_*, *r_n_*, *z_nk_* and *c_nk_*) without violating the associated constraints. **Step 4) Calculate** the objective value of the problem (IP).

The following section will show a complete algorithm (denoted *LGR*), as based on subgradient method [1] for solving problem (IP). The computational complexity of the LGR is *O*(|*S*||*N*|^2^ + |*N*||*R_n_*| *T*).

**Algorithm 2.** LGR Algorithm.

**Begin**
*Input*: Network topology, data source nodes
*Output:* Data aggregation tree
*Initialize* Lagrangean multiplier vectors *u^i^* (0) = 0, ∀*i* = 1,…,6.
UB = ∞ and LB = −∞ (upper and lower bounds, respectively).
*quiescence_age* = 0, and *step_size* = 2.
**For***iteration****=*** 1 to *Max_Iteration*, perform the following:
*Solve*subproblem 1, subproblem 2, subproblem 3, subproblem 4.
*Compute Z_D_* in (LR).
**If***Z_D_* (*u*) > *LB*
*LB* = *Z_D_* (*u*) and *quiescence_age* = 0.
**Else***quiescence_age = quiescence_age* + 1.
**If***quiescence_age* = *Quiescence_Threshold*
*step_size = step_size/2* and *quiescence_age* = 0.
*Run*Algorithm 1 (*LGR-Primal*). Compute the new upper bound *ub*.
**If***ub* < UB then UB = *ub*.
*Update* the *step_size*.
*Update* the Lagrangean multiplier vectors.
**End For**
**End**

## 6. Computational Experiments

The proposed algorithms for MAC-DAR problems are coded in C and run on a PC with PIV-2G. In a LGR algorithm, *Max_Iteration* and *quiescence_age* are set to 2000 and 30, respectively. The step size coefficient *step_size*, is initialized as 2, and is halved when the objective function value of the dual problem is not improved by iterations reaching *quiescence_age*. The computational times for the following experiments are all within five minutes.

**Figure 3.**
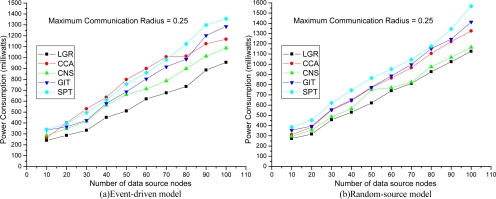
Total Energy Consumption with respect to no. of data source nodes.

**Figure 4.**
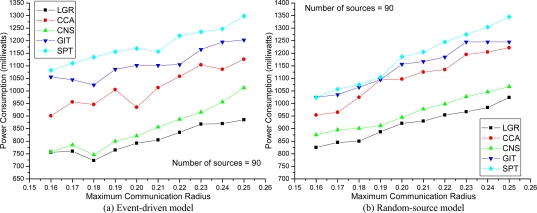
Total Energy Consumption with respect to Transmission Radius.

**Figure 5.**
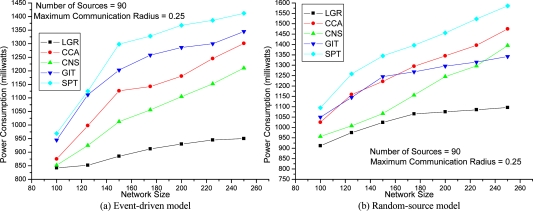
Total Energy Consumption with respect to Network Size.

The network topology comprises |*N*| (= 150 in Figure 3 and 4, up-to 250 in Figure 5) sensor nodes randomly placed within a 1×1 square unit area. The cost of the energy consumption function (in milliwatts), *e_n_* (*r_n_*), is defined as the square of 100×Euclidean distance multiplied by the energy consumption per millisecond when the sensor node is transmitting data. The set of all possible transmission radii of sensor node *n* (i.e., *R_n_*) are configured to begin from 0 to the *maximum communication radius* (e.g. 0.25 in Figure 3) with step size 0.01. The CSMA/CA related parameters (*RTS*, *SIFS*, *θ*) are the same settings as in [4]. To evaluate the solution quality of our proposed algorithm, four existing algorithms are implemented for comparison. The SPT, GIT, and CNS algorithms are proposed in [2], and the forth algorithm CCA, is described in [5]. It is worth noting that, all four heuristic constructs data aggregation trees without considering MAC layer collision effects. Each plotted point in Figures 3–5 is a mean value over 10 simulation results.

Two different models in WSN are simulated. The first is an *event-driven*, where neighboring sensor nodes of the event will become the data source nodes. The second is a *random-source*, where data source nodes are determined in random. Hence, the data source nodes in an event-driven model will be closer to each other than in a random-source model.

Figure 3 shows the total energy consumption with increasing numbers of data source nodes. When the number of source nodes is large, the aggregation tree is larger. It is shown that the LGR algorithm can obtain the best solution quality, as compared to other heuristics in both event-driven and random-source models. In addition, in an event-driven model, the solution quality of LGR algorithm is even more significant than other heuristics. This is because in an event-driven model, the data source nodes are clustered to increase probability of collisions. Hence, heuristic algorithms that do not address the MAC collision suffer from severe retransmission occurrences.

In Figure 4, the effects of communication radii on energy consumption are examined with 90 data source nodes. As shown, the LGR algorithm can still obtain best solution quality, as compared to the other heuristics on both models. Interestingly, even though a large maximum communication radius could increase the probability of data aggregation, it is shown that a large maximum communication radius did not offer any advantage for MAC aware energy efficient data aggregation trees, because a large communication radius leads to severe collisions that could jeopardize the advantages of data aggregation. In addition, in an event-driven model, the best solution for the LGR algorithm is at 0.18, instead of at 0.16 communication radius. This reveals that; for too small communication radii, even though the collision probability is low, it will not provide data aggregation advantages that save total transmission power. Hence, best communication radii setting should consider the tradeoff between collision and data aggregation.

Figure 5 depicts the experiments evaluating the solution quality under different network sizes (i.e., network density). The LGR algorithm outperforms the other heuristics for all the network sizes. In large network size (i.e., a high density of sensor nodes within a fixed deployment area), the solution quality of LGR over the other heuristics is more significant. Recall that in the first term (i.e., $\text{min}\;\sum_{n\in N}t_{\mathrm{data}}\cdot e_{n}(r_{n})$) of the objective function in problem (IP), which favors communication links of shorter distances, thus, the data aggregation tree will be composed of more relay nodes for data aggregation. However, in this case, as too many relay nodes on the data aggregation tree will introduce higher probability of collision, we will have larger retransmission cost (i.e., the second term of the objective function in problem (IP)). It is expected that the second term will play a more important role in a dense network topology. According to Figure 5, the solution for LGR increase more mildly than the other heuristics in a dense network topology, which reveals that LGR will not select a large number of hops for data aggregation, in order to avoid extra energy loss from retransmissions in a dense network topology.

The improvement ratio is defined as (other approach — LGR)/(LGR)×100% to show the solution quality. In Table 1, the improvement ratio of LGR over SPT, GIT, CNS, and CCA is 57%, 42%, 29%, and 59%, respectively.

## 7. Conclusions

In addition to the data aggregation, retransmission energy loss due to MAC collision plays another major crucial factor for energy-efficient data-aggregation routing in WSNs. This paper is the first one proposing a novel nonlinear mathematical formulation for MAC aware energy efficient data aggregation routing problems in WSN, where the objective function is to minimize the total energy consumption (including data transmission power and retransmission power) subject to data aggregation trees, transmission coverage, and data retransmissions constraints. The proposed solution approach is based on Lagrangean relaxation to construct a MAC aware energy-efficient data aggregation tree that jointly considers the tradeoff between data aggregation and data retransmission by using the Lagrangean multipliers. According to the computational experiments, the proposed LGR algorithm outperforms other heuristics under all tested cases, especially in an event-driven model. This is because in an event-driven model, the data source nodes are clustered, and thus, extra energy loss from retransmissions will be more significant. This indicates that a good data aggregation algorithm should be a cross layer algorithm that jointly addresses data aggregation in the network layer, and the retransmission energy loss in the MAC layer.

## Figures and Tables

**Table 1.** Improvement Ratio.

**Improvement Ratio**	Figure 3	Figure 4	Figure 5
SPT	(53%, 43%)	(57%, 33%)	(49%, 45%)
GIT	(40%, 30%)	(42%, 29%)	(42%, 22%)
CNS	(29%, 21%)	(14%, 6%)	(27%, 27%)
CCA	(59%, 24%)	(31%, 24%)	(37%, 35%)
